# Hepatobiliary organoids differentiated from hiPSCs relieve cholestasis-induced liver fibrosis in nonhuman primates

**DOI:** 10.7150/ijbs.90441

**Published:** 2024-01-21

**Authors:** Hongmei Li, Jingyi Li, Ting Wang, Ke Sun, Guangrui Huang, Yulin Cao, Fenfang Wu, Anlong Xu

**Affiliations:** 1 School of Life Science, Beijing University of Chinese Medicine, Beijing 100029, People's Republic of China.; 2 Beizhong Jingyuan Biotechnology (Beijing) Limited, Beijing, People's Republic of China.; 3 Shenzhen Hospital, Beijing University of Chinese Medicine, Shenzhen 518116, People's Republic of China.; 4 Tangyi Holdings (Shenzhen) Limited, Shenzhen, People's Republic of China.; 5 State Key Laboratory of Biocontrol, Guangdong Province Key Laboratory for Pharmaceutical Functional Genes, College of Life Sciences, Sun Yat-Sen University, Guangzhou, Guangdong 510006, People's Republic of China.

**Keywords:** HBOs induced from hiPSCs, Liver subcapsular transplantation, Submesenteric transplantation, Safety and efficacy, Hepatoprotective effect and mechanism

## Abstract

There is an urgent need for novel therapies to treat end-stage liver disease due to the shortage of available organs. Although cell transplantation holds considerable promise, its availability is limited due to the low engrafted cell mass and lack of unifying cell transplantation strategies. Here, we optimally established human induced pluripotent stem cell-derived functional hepatobiliary organoids (HBOs) based on our previous research and transplanted them into a monkey model via liver subcapsular and submesenteric transplantation routes to assess their potential clinical application. Our study revealed that HBO transplantation could safely and effectively improve hepatoprotection effects by antiapoptotic and antifibrotic agents. In addition, we also discovered that while multiple HBO transplantation pathways may have a shared effector mechanism, their respective treatment approaches have distinct advantages. Transplantation of HBOs could promote the high expression of CTSV in hepatic sinusoid endothelial cells, which might halt the progression of hepatic sinusoidal capillarization and liver fibrosis. Liver subcapsular transplants had stronger pro-CTSV upregulation than HBO submesenteric transplants, which could be attributed to naturally high CTSV expression in HBOs. Interestingly, both transplantation routes of HBOs were targeted the injured liver and triggered a new pattern of ductular reaction to alleviate the degree of liver fibrosis by surrounding the area with CK19-positive labeled cells. These residing, homing and repairing effects might be related to the high expression of MMP family genes. By promoting a unique pattern of ductular reactions, submesenteric HBO transplantation has a more representative antifibrotic impact than liver subcapsular transplantation. Altogether, our data strongly imply that the treatment of severe liver diseases with liver subcapsular and submesenteric transplantation of HBOs may be clinically effective and safe. These findings provide new insight into HBOs for further experimental and clinical validation.

## Introduction

Due to the lack of efficient therapy, liver fibrosis is a major health concern that results in high morbidity and mortality 1. Hepatic fibrosis can be caused by a number of conditions, including viral infections, acute hepatitis, cholestasis, metabolic disorders, alcohol abuse and autoimmunity 2. If left untreated, long-term liver fibrosis can progress into cirrhosis, hepatic failure and hepatocellular carcinoma 3. To date, there is no pharmacological treatment for liver fibrosis 4. Liver transplantation is the main curative clinical therapy for end-stage liver disease, but the scope of this treatment is limited by the shortage of donor livers for transplantation. Therefore, the development of novel regenerative therapies is urgently needed.

With the advancement of stem cell therapy, stem cells have become a hot topic in the field of liver regeneration research. At present, there have been clinical studies on the treatment of liver diseases with hematopoietic stem cells or mesenchymal stem cells, and some progresses have been made 5-8. Although mesenchymal stem cell transplantation cannot completely cure liver disease, it can improve liver function and long-term survival in decompensated cirrhosis. In recent years, a variety of tissue organoids have been derived from patient-specific pluripotent stem cells and show promise as a potential cell source for organoid transplantation. Relevant literature on liver organoid transplantation in rodents has been reported 10, 11, confirming that human liver organoids can proliferate in the livers of mouse models. The results of Fotios et al. suggested that cholangiocyte organoids could be used to repair human biliary epithelium 12. Additionally, bile cholangiocyte organoids efficiently repopulate decellularized extrahepatic bile duct scaffolds and restore the monolayer of cholangiocyte-like cells *in vitro* 13. The above studies have provided multiple lines of evidence for liver repair by liver organoids and bile duct organoids, but the safety and efficacy of hepatobiliary organoid (HBO) transplantation with hepatobiliary dual-lineage characteristics to repair the liver has not yet been reported.

Furthermore, the selection of suitable transplantation sites has become an important topic for the clinical translation of organoids. Because of the risk of endovascular embolism, organoids are not suitable for intravenous transplantation, and the digestion of organoids into single-cell suspensions for transplantation may damage their structural integrity and functional stability. To date, studies have reported multiple sites of liver organoid transplantation, including the liver orthotopic 14, 15, portal vein 16, mesentery 17, cranial window 18, renal capsule 19, omentum 20, etc., but there is still a lack of studies on the comparative analysis of the efficacy of multiroute organoid transplantation in nonhuman primate models.

To address these issues, our team has developed a technique that allows human induced pluripotent stem cells (hiPSCs) to produce functional HBOs without the use of exogenous cells or genetic manipulation by adding specially prepared cholesterol^+^MIX, which is primarily made up of cholesterol and other small molecules 21. Considering that the optimally induced HBOs displayed both hepatic and biliary functional attributes, we predicted that HBOs could exert a better therapeutic effect* in vivo*. To confirm the safety and effectiveness of HBO transplantation, we chose the liver and mesentery as the two transplantation sites to investigate the efficacy and mechanism variations of different transplantation routes of HBOs on cholestasis-induced liver fibrosis in nonhuman primates. This study revealed the crucial role and underlying therapeutic mechanisms of HBO transplantation in treating liver fibrosis. The high expression of MMP family genes may contribute to the ectopic homing and *in situ* residence of HBOs, and more importantly, the CK19-positive labeling of a new ductular reaction pattern and the high expression of cathepsin V (CTSV) may serve as potential therapeutic targets for liver fibrosis.

## Results

### Characterization of optimally induced HBOs for transplantation

The hiPSCs used in our study were cultured on Matrigel-coated plates prior to differentiation and were observed under a microscope to evaluate the formation of compact colonies with clear boundaries (Figure S1A). The hiPSCs stained consistently positive for the pluripotent stem cell-specific markers OCT4 and NANOG (Figure S1B). Flow cytometry results showed that the proportion of hiPSCs with double-positive expression of OCT4 and NANOG was as high as 96.2%, indicating that hiPSCs had good pluripotency, which could support the differentiation of HBOs and long-term culture *in vitro* (Figure S1C). Next, hiPSCs were differentiated into HBOs using the protocol independently developed by our research team 21 (Figure S2A). Under light microscopy, the HBOs on the 15th day of differentiation morphologically changed into polygonal hepatocellular cells with an indistinct border, indicating that they were mostly in the liver progenitors stage (Figure S2B). By Day 35, the angular hexagonal hepatocytes in the lower layer and the vesicle-like biliary lineage cells in the upper layer were signs that HBOs had matured (Figure S2C, D). HBOs exhibited biliary cell features that were positively stained for CK19 by immunohistochemistry (Figure S2E). The results of immunofluorescence staining showed that mature HBOs could simultaneously express the liver cell markers ALB and CYP3A4 and contained CD31-positive vascular endothelial structures. All cells in HBOs did not express pluripotency markers, as indicated by immunofluorescence co-staining of HBO slices with the stem cell pluripotency markers OCT4 and NANOG (Figure S2G). This result suggested that mature HBOs had little chance of teratoma when utilized for *in vivo* transplantation. Oil Red O staining of HBOs showed that most of the cells contained red lipid droplets, indicating that liver cells in HBOs had a lipid storage function similar to that of human hepatocytes (Figure S2F). The content of ALB in the culture supernatant was measured to identify the secretory function of HBOs. ELISA results showed that HBOs already exhibited ALB secretory function on Day 26 of differentiation, reaching the highest level on Day 34 to 36 and exhibited a slight decrease from Day 38 (Figure S2H). Therefore, we selected the HBOs with the strongest secretory function on the 35th day of differentiation for the following *in vivo* transplantation experiment.

### The protective effect of HBO transplantation on liver enzymes in cholestasis-induced liver fibrosis monkeys

**Figure 1:**
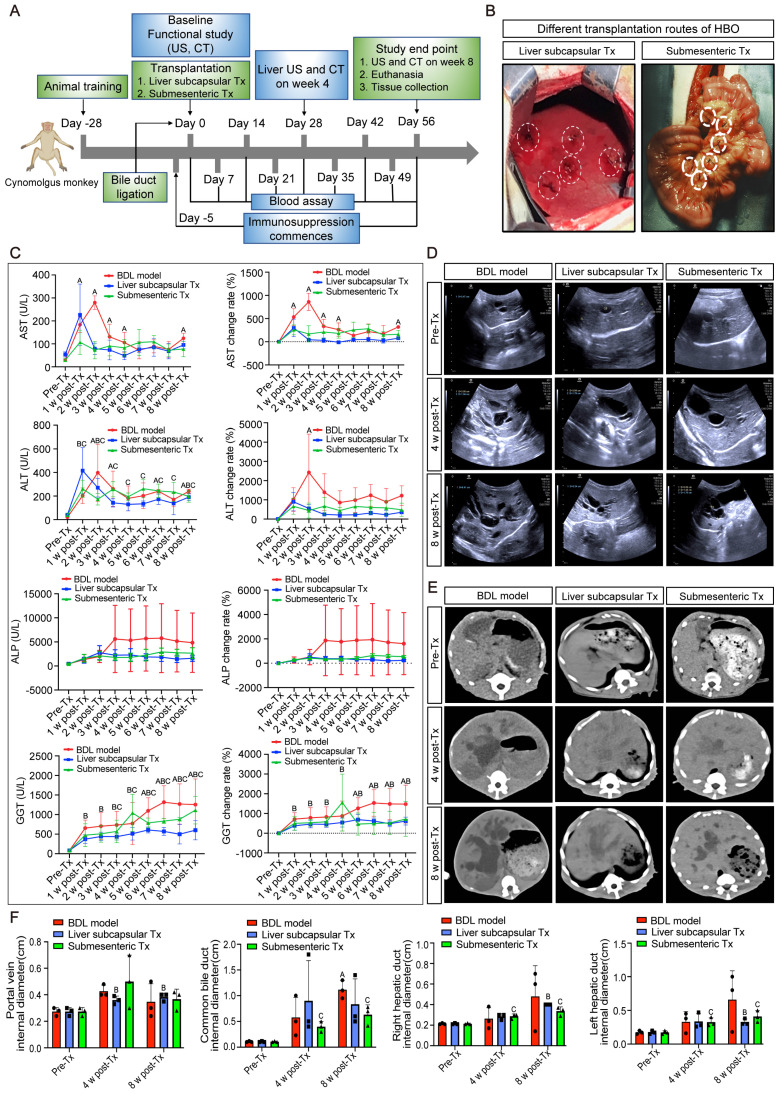
** Radiographic and biochemical evaluation of HBO transplantation therapy. (A)** Timeline of transplantation and experimental sessions for liver fibrosis model of nonhuman primates. The timeline includes animal training, experimental treatment phases and sacrifice points. **(B)** Schematic diagram of the liver subcapsular and submesenteric transplantation procedure. **(C)** Changes of biochemical parameters in nonhuman primates at different time points after HBO transplantation. AST, aspartate aminotransferase; ALT, alanine aminotransferase; ALP, alkaline phosphatase; GGT, gamma-glutamyl transferase. n = 3 cynomolgus monkeys per group. ^A^ indicates *P*<0.05 post-Tx versus pre-Tx in the BDL model group. ^B^ indicates *P*<0.05 post-Tx versus pre-Tx in the Liver subcapsular Tx group. ^C^ indicates *P*<0.05 post-Tx versus pre-Tx in the Submesenteric Tx group. **(D)** Ultrasonographic representation images of the liver in transverse section. n = 3 cynomolgus monkeys per group. **(E)** Axial computed tomography (CT) images of the lesion in the liver. **(F)** Quantification of changes in hepatic vascular diameter after HBO transplantation. n = 3 cynomolgus monkeys per group. Mean ± SEM. ^A^ indicates *P*<0.05 post-Tx versus pre-Tx in the BDL model group. ^B^ indicates *P*<0.05 post-Tx versus pre-Tx in the Liver subcapsular Tx group. ^C^ indicates *P*<0.05 post-Tx versus pre-Tx in the Submesenteric Tx group. BDL model, bile duct ligation fibrosis model group without HBO transplantation; Liver subcapsular Tx, liver orthotopic transplantation group; Submesenteric Tx, heterotopic submesenteric transplantation group; Pre-Tx, before modeling and transplantation; 1 w post-Tx, 1 week after modeling and transplantation; 2 w post-Tx, 2 weeks after modeling and transplantation; 3 w post-Tx, 3 weeks after modeling and transplantation; 4 w post-Tx, 4 weeks after modeling and transplantation; 5 w post-Tx, 5 weeks after modeling and transplantation; 6 w post-Tx, 6 weeks after modeling and transplantation; 7 w post-Tx, 7 weeks after modeling and transplantation; 8 w post-Tx, 8 weeks after modeling and transplantation.

In the investigation on *in vivo* transplantation of HBO, bile duct ligation modeling was carried out. Five days prior to the transplantation of HBO (referred to as Tx), immunosuppressive injections were initiated. The day following modeling, HBO was transferred via liver subcapsular and submesenteric routes (Figure 1A, B). Before modeling, blood was drawn from each monkey (Pre-Tx), and at Days 7, 14, 21, 28, 35, 42, 49 and 56 after liver subcapsular or submesenteric transplantation of HBOs, liver injury was assessed by determining the serum levels of the liver enzymes. The results showed that the aspartate aminotransferase (AST) content of the bile duct ligation (BDL) fibrosis model group increased gradually after modeling, peaked in the second week, and was approximately 10 times higher than that prior to modeling. From the third week on, the AST level in the BDL model group dropped to four times the pre-modeling level and plateaued until the eighth week. In the submesenteric Tx group, the AST level remained 3-4 times higher than that before modeling from 1 to 8 weeks after transplantation and did not show a progressive increase in the period of acute liver injury. Due to the liver damage caused by the liver subcapsular transplantation route, AST initially increased temporarily 1 week after HBO transplantation but rapidly decreased in the second week and remained low until the eighth week. Another enzyme, alanine aminotransferase (ALT), followed the same trend as AST levels.

Then, alkaline phosphatase (ALP) and gamma-glutamyl transferase (GGT), the blood indicators for cholestatic liver damage, were assessed. Three weeks after the modeling period, the level of ALP increased rapidly and maintained a high value for 8 weeks. The level of ALP following HBO transplantation tended to be steady in comparison to that in the BDL model group, and the expression of ALP in the liver subcapsular Tx and submesenteric Tx groups did not exhibit a discernible upward trend. For the level of GGT, the trend of change in the HBO transplantation group and BDL model group was essentially the same within 5 weeks after modeling, but the BDL model group gradually increased from the fifth week and maintained the highest level from the sixth to the eighth week, close to approximately 15 times the level of GGT before modeling. Compared with that in the BDL model group, the GGT level in the liver subcapsular Tx group did not change much from week 6 to week 8, while it fluctuated slightly in the submesenteric Tx group. Even so, the elevated GGT levels were still lower than those in the BDL model group. These data demonstrated that HBO transplantation had a protective effect against elevated liver enzymes induced by cholestasis in monkeys (Figure 1C).

### Abdominal ultrasound and CT scans demonstrated that HBO transplantation had the potential to alleviate liver cholestasis

CT and abdominal ultrasound scans were performed prior to, four weeks after, and eight weeks after modeling. The results of the ultrasound revealed that the surface of the monkey's liver was smooth and had a homogenous parenchyma echo before the procedure. The common bile duct, portal vein, and hepatic ducts did not appear to be dilated. The echo of the monkey liver in the BDL model group, liver subcapsular Tx, and submesenteric Tx groups at 4 and 8 weeks after modeling was obviously enhanced. Scattered pierced punctured strong echoes, dilated portal veins, and common bile ducts as well as hepatic ducts were observed simultaneously. However, at 8 weeks following transplantation, the levels of common bile duct and left hepatic duct dilatation in the subcapsular Tx and submesenteric Tx groups' livers were lower than those in the BDL model group (Figure 1D, F). According to the CT scan, the BDL model group's liver volume was increased, the intrahepatic and extrahepatic bile ducts were noticeably dilated, and the low-density cholestasis shadows of the liver parenchyma were progressively enlarged. Compared with the BDL model group, the proportion of the liver parenchyma with a low-density shadow region was dramatically reduced in the liver subcapsular Tx and submesenteric Tx groups, and the degree of intrahepatic and extrahepatic bile duct dilatation was relatively mild (Figure 1E). The results showed that HBO transplantation, by reducing cholestasis and enhancing the condition of the injured liver, could alleviate compensatory bile duct and hepatic duct dilatation.

### HBO transplantation could efficiently and safely prevent splenomegaly caused by cholestatic liver injury

**Figure 2:**
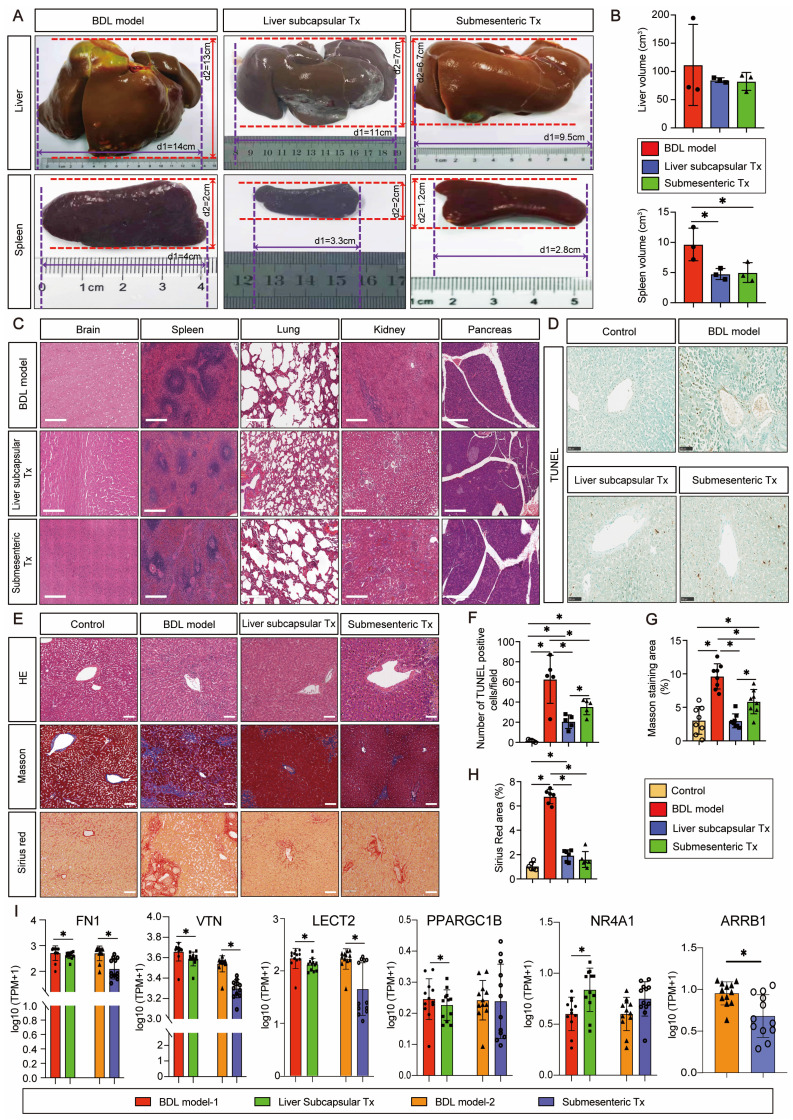
**HBO transplantation attenuated liver fibrosis and apoptosis. (A-B)** Representative plot of liver and spleen from BDL model, and HBOs transplanted monkeys (A), quantitative analysis of organ sizes. (B) Comparisons were shown. * Indicates *P*<0.05. n = 3 monkeys for model, liver subcapsular and submesenteric Tx group. **(C)** Histologic evaluation of HE stained paraffin-embedded tissue sections. HE-staining revealed no evidence of tumor formation after HBO transplantation. Scale bars = 400 μm. **(D)** Apoptosis in the liver sections was assessed by TUNEL assay, which indicating the DNA strand breaks in fibrotic livers. Scale bar = 100 μm. **(E)** The extent of fibrosis was examined by staining paraffin-embedded liver sections using Masson's trichrome and Sirius Red staining. Representative photomicrographs of HE, Masson (blue-stained area) and Sirius Red (red-stained area) staining demonstrated the presence of collagen fibers in slides. Scale bars = 100 μm. **(F)** Statistical results of TUNEL-positive cells per field (n=5). Mean ± SEM. ^*^P < 0.05.** (G)** Fibrosis was assessed by semi-quantification of Masson trichrome-positive tissue with the Image-Pro plus analysis system. * Indicates *P*<0.05. **(H)** Quantification of Sirius Red areas in the liver sections calculated by dense intensity of Sirius Red relative to the amount of background per field in 6 randomly chosen microphotographs. Mean ± SEM. ^*^P < 0.05. Control, untreated healthy monkey; BDL model, bile duct ligation fibrosis model group without HBO transplantation; Liver subcapsular Tx, liver orthotopic transplantation group; Submesenteric Tx, heterotopic submesenteric transplantation group. **(I)** Based on RNA sequencing study of hepatic fibrosis pathological regions in BDL model-1 vs. liver subcapsular and BDL model-2 vs. submesenteric Tx groups, the expression values of six liver fibrosis-related genes (FN1, VTN, LECT2, PPARGC1B, NR4A1 and ARRB1) were presented in log10 1+TPM. FN1, fibronectin 1; VTN, vitronectin; LECT2, leukocyte cell-derived chemotaxin 2; PPARGC1B, peroxisome proliferator-activated receptor gamma coactivator 1 beta; NR4A1, nuclear receptor subfamily 4 group A member 1; ARRB1, arrestin beta 1; BDL model-1, bile duct ligation fibrosis model group without HBO transplantation for comparison with liver subcapsular Tx group; Liver subcapsular Tx, liver orthotopic transplantation group; BDL model-2, bile duct ligation fibrosis model group without HBO transplantation for comparison with submesenteric Tx group; Submesenteric Tx, heterotopic submesenteric Tx group.

Monkeys were sacrificed after 8 weeks of modeling and transplantation, and the entire liver and spleen were removed to examine the gross morphology. After liver subcapsular and submesenteric transplantation of HBOs, we observed that there was no teratoma formation in the liver or spleen. The liver capsule of the BDL model group had a hard texture with yellow protrusions on the surface, whereas the liver capsule of the HBO transplantation groups was smooth and complete, with a somewhat flexible texture that maintained the relatively normal morphology of the liver and spleen (Figure 2A). The quantitative statistics of liver and spleen volumes revealed that the model group had a significantly larger spleen volume than the HBO transplantation groups (*P* < 0.05), but there was no significant difference in spleen and liver volumes between the liver subcapsular Tx and submesenteric Tx groups (Figure 2B). Then, we further selected key organs of experimental monkeys for HE staining to identify the safety of HBO transplantation *in vivo*. There was no evidence of teratoma formation in the brain, spleen, lung, kidney, or pancreas, confirming the safety of potential treatment (Figure 2C). Additionally, animal plasma was isolated to detect renal function indices such as UREA and CREA (Figure S3A, B), cardiac sensitive indices such as CK and LDH (Figure S3C, D), routine blood indices such as NEUT and LYMPH (Figure S3E, F), and coagulation function indices such as APTT, PT, and Fbg in each group (Figure S3G-I). There were no abnormal elevation values, which laterally confirmed the systemic safety of HBO transplantation therapy. Furthermore, we tracked changes for the total bilirubin (TBIL) index and found no statistically significant difference between the three groups at any particular time point following transplantation. However, at 3-8 weeks post-transplantation, the submesenteric Tx group's TBIL values were significantly higher than the preoperative baseline values, even though the rate of change did not differ statistically (Figure S3J).

### Cholestasis-related apoptosis and fibrosis could be alleviated by HBO transplantation

By *in situ* labeling with the TUNEL assay, apoptotic cells in liver slices were found. Representative pictures indicate that the BDL model group was more likely to exhibit significant apoptosis and positive TUNEL staining in the nuclei than the control group after 8 weeks of modeling (Figure 2D). Quantitative statistical analysis of apoptotic cells revealed that (Figure 2F), in comparison to the BDL model group, the apoptotic cells in the HBO transplantation groups were greatly decreased, and the antiapoptotic effect in the liver subcapsular Tx group was more significant than that in the submesenteric Tx group (*P* < 0.05). According to the HE staining results, livers from the control group had normal lobular architectures, whereas livers from the BDL model group had damaged lobular architectures, severe hepatocyte vacuolar degeneration, extensive fibrous septa, pseudolobule forms, and inflammatory cell infiltration, all of which were significantly improved by HBO transplantation. Masson and Sirius Red staining provided additional evidence to support these findings (Figure 2E). The liver tissues from the control group had little collagen deposition, but those from the BDL fibrosis model group had dense fibrous septa and elevated collagen deposition. As shown by quantitative analysis of liver injury and fibrosis, HBO transplantation considerably reduced the liver injury and fibrosis caused by cholestasis (Figure 2G, H). To confirm the effect of HBO transplantation on the expression of fibrosis-related genes, we performed RNA sequencing on liver lesion tissues and discovered that three liver fibrosis-sensitive indicators, FN1, VTN, and LECT2, were significantly downregulated after both liver subcapsular and submesenteric HBO transplantation. In contrast, there was no statistically significant difference between PPARGC1B and NR4A1 in the submesenteric Tx group. These two other sensitive indicators were only significantly regulated in the liver subcapsular Tx group (*P*<0.05).

Additionally, ARRB1 was only significantly downregulated in the submesenteric Tx group, with no detectable expression of this gene in the liver subcapsular Tx group (Figure 2I). All of these findings confirm that HBOs have protective properties against hepatic fibrosis and apoptosis.

### Transplantation of HBOs inhibited capillarization of liver sinusoids and upregulated CTSV gene expression

**Figure 3:**
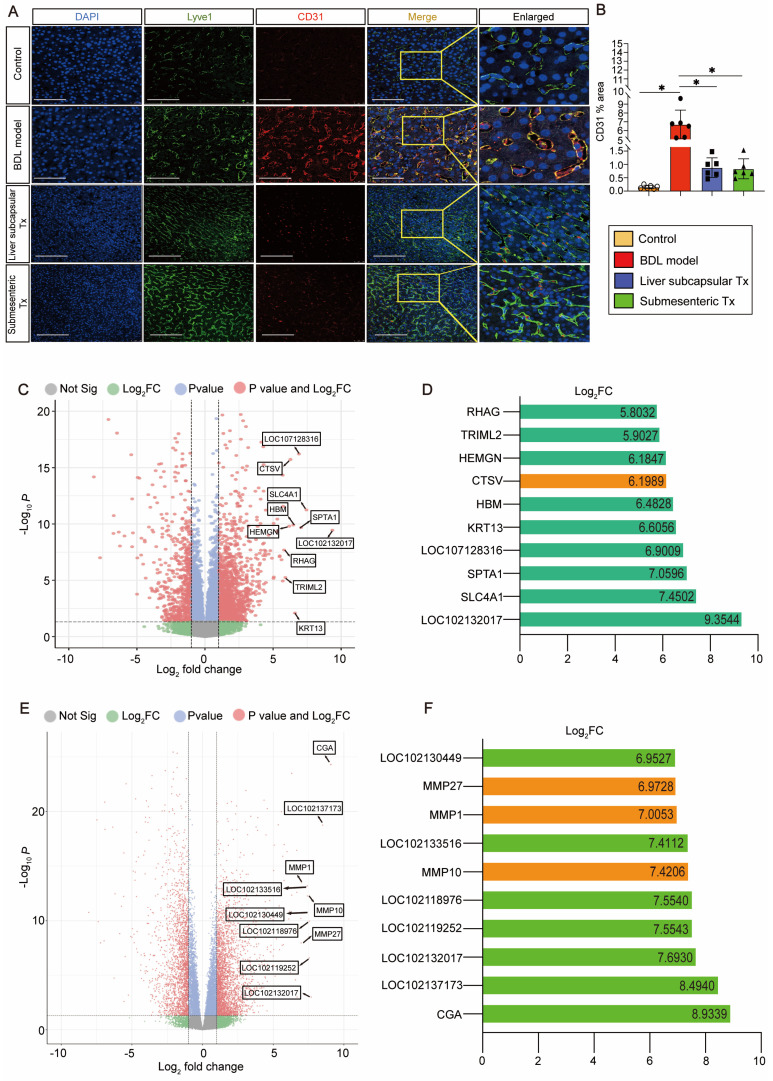
** HBO transplantation decreased sinusoid capillarization and affected gene expression in the liver. (A)** Lyve1 and CD31 immunostaining were used to locate and evaluate the degree of capillarization of hepatic sinusoids. Lyve1: green, CD31: red. Scale bars = 75 μm. **(B)** Quantification of CD31-positive area per field (n=6). Mean ± SEM. ^*^P < 0.05.** (C)** Genome-scale differential expression levels of mRNA between the BDL model and liver subcapsular Tx group were screened using a volcano plot. **(D)** Ranking of the top 10 up-regulated genes in the liver following liver subcapsular transplantation of HBOs, based on RNA sequencing (RNA-seq) analysis. Among the up-regulated genes, we noticed the gene CTSV, which can participate in matrix degradation and cell invasion. **(E)** Volcano plots for the RNA-seq analyses of BDL model and submesenteric Tx monkey liver samples. **(F)** Ranking of the top 10 up-regulated genes in the liver following submesenteric transplantation of HBOs, based on RNA-seq analysis. Among 10 of the up-regulated genes, we noticed a group of MMPs genes (MMP10, MMP1 and MMP27) related to cell homing. Log_2_FC, Log 2-fold change. -log_10_*P*, negative logarithm of the *P* value. Control, untreated healthy monkey; BDL model, bile duct ligation fibrosis model group without HBO transplantation; Liver subcapsular Tx, liver orthotopic transplantation group; Submesenteric Tx, heterotopic submesenteric transplantation group.

We discovered an intriguing phenomenon whereby transplantation of HBOs could reverse liver sinusoidal capillarization. The immunofluorescence method was used to double stain Lyve-1, a marker of liver sinusoid endothelial cells (LSECs) 22, and CD31, a marker of LSEC capillarization 23. We found that Lyve-1-positive LSECs did not co-express CD31-positive capillaries in the normal control liver, and a significant amount of CD31-positive LSEC expression appeared in the injured liver after modeling, but the pathological process of LSEC capillarization was significantly reversed after liver subcapsular and submesenteric transplantation of HBOs (Figure 3A, B). Next, we conducted RNA-Seq analysis on the livers from each group of monkeys to further investigate the internal mechanism of liver subcapsular and submesenteric transplantation of HBOs to reverse LSEC capillarization. We screened out the top 10 genes with a log2-fold change in the differential genes between the liver subcapsular Tx group and the BDL model group and noticed that the expression of CTSV was considerably upregulated after liver subcapsular transplantation of HBOs. There is, however, no comprehensive literature demonstrating a link between the other nine elevated genes and liver injury healing (Figure 3C, D). Then, we sequenced the transcripts from the BDL model group and submesenteric Tx group of monkey livers and analyzed the differentially expressed genes between the two groups. A total of 4234 differentially expressed genes, including 2473 highly upregulated genes and 1911 significantly downregulated genes, were selected by comparing the sequencing data of the BDL model group with the submesenteric Tx group. According to our analysis of the enriched differential genes, the top 10 genes that were highly upregulated in the submesenteric Tx group were CGA, LOC102137173, LOC102132017, LOC102119252, LOC102118976, MMP10, LOC102133516, MMP1, MMP27 and LOC102130449 (Figure 3E). Because the whole-genome sequencing of the reference species, the cynomolgus monkey, has not been published or documented, some genes are named after LOC. Among the top ten significant genes, MMP10, MMP1, and MMP27 were found to be members of the MMP family, which may play an important role in contributing to the fact that HBOs can be retained or can nest home to the injured liver after transplantation (Figure 3F).

Drawing from the aforementioned observations, we deduce that following an HBO transplant, HBO-detached cells have the ability to live *in situ* within the liver or migrate there with the assistance of MMP family members. Additionally, they can safely and effectively reverse the capillarization of pathogenic LSECs and induce high expression of CTSV genes, which are thought to play a protective role in the liver.

### With minimal perisinusoidal fibrosis, LSECs showed high expression of CTSV induced by transplantation of HBOs

**Figure 4:**
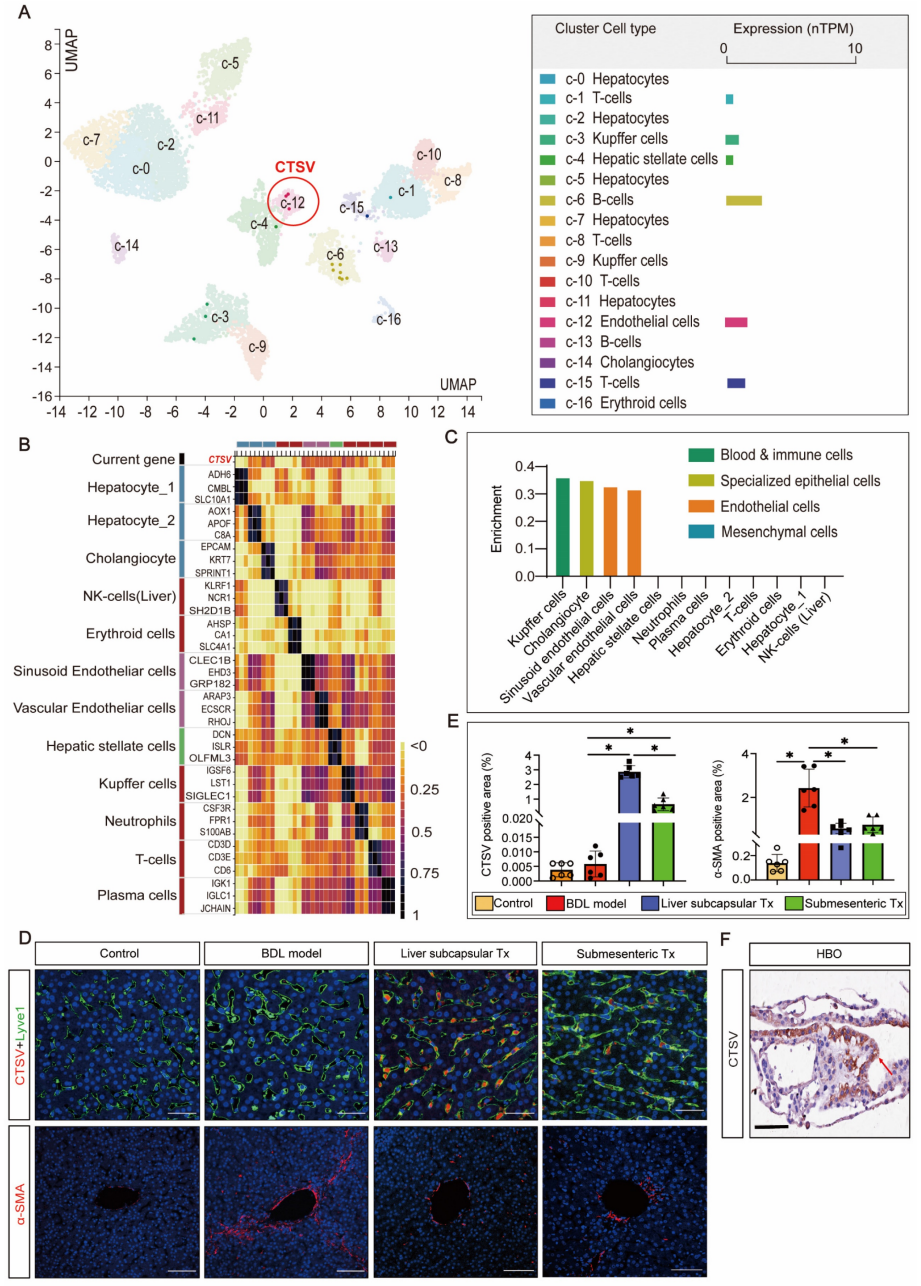
** The high expression of CTSV induced by HBO transplantation existed in LSECs and it helped to improve perisinusoidal fibrosis. (A-C)** Analysis of gene expression of CTSV was obtained from the single cell type and tissue cell type sections in the HPA database. The results showed that CTSV was highly correlated with hepatic sinusoid endothelial cells and could be expressed in these cells. **(D)** Immunofluorescence staining for the CTSV, Lyve1 and α-SMA in liver tissue sections after 8 weeks of HBO transplantation treatment. Representative micrographs demonstrated increased presence of CTSV and decreased expression of α-SMA in HBOs Tx groups compared to BDL model group. Scale bar = 50 μm. **(E)** Quantification of CTSV and α-SMA immunofluorescence intensity with IPP software. Six visual fields were randomly selected. All data were represented as the mean ± SD. ^*^P<0.05 was considered significant. **(F)** Representative immunohistochemical image of CTSV-positive cells in HBOs. The cell that stains positively for CTSV is located in the brown area that is indicated by the red arrow. The results revealed that CTSV was highly expressed in the HBOs used for transplantation. Scale bar = 100 μm. Control, untreated healthy monkey; BDL model, bile duct ligation fibrosis model group without HBO transplantation; Liver subcapsular Tx, liver orthotopic transplantation group; Submesenteric Tx, heterotopic submesenteric transplantation group.

Since there is no published research on the relationship between CTSV and liver sinusoid capillarization and fibrosis, single-cell sequencing data from the HPA database were searched to cluster the expression of CTSV in different types of liver cells. The results showed that B cells and endothelial cells exhibit the highest levels of CTSV expression (Figure 4A). The relationship between CTSV and the classification of endothelial cells was then further defined. CTSV could be significantly expressed in hepatic sinusoid endothelial cells and vascular endothelial cells, according to heatmap analysis and relative expression data between the CTSV gene and other liver cells (Figure 4B, C).

To confirm the impact of HBO transplantation on the expression of CTSV in LSECs and perisinusoidal fibrosis, we carried out Lyve-1 and CTSV fluorescence co-staining and α-SMA fluorescence single staining on liver sinusoidal tissue slices. The outcomes demonstrated that liver subcapsular and submesenteric transplantation of HBOs significantly enhanced the expression of CTSV in LSECs, whereas the degree of hepatic fibrosis shown by α-SMA fluorescence was inversely reduced with the high expression of CTSV, and this modulatory effect was more pronounced in the liver subcapsular transplantation group (Figure 4D,E). Furthermore, we examined the expression of CTSV in HBO that was utilized for transplants and were surprised to see that HBO expresses a significant amount of CTSV (Figure 4F). These findings together suggested that the increased expression of CTSV in LSECs may play a role in the capacity of HBO transplantation to reverse hepatic sinusoidal capillarization and hepatic fibrosis and that the inherently high expression of CTSV in HBOs may be somewhat connected to the upregulated expression of CTSV in the liver.

### Transplantation of HBOs induced the formation of a new DR pattern and slowed the progression of liver fibrosis

**Figure 5:**
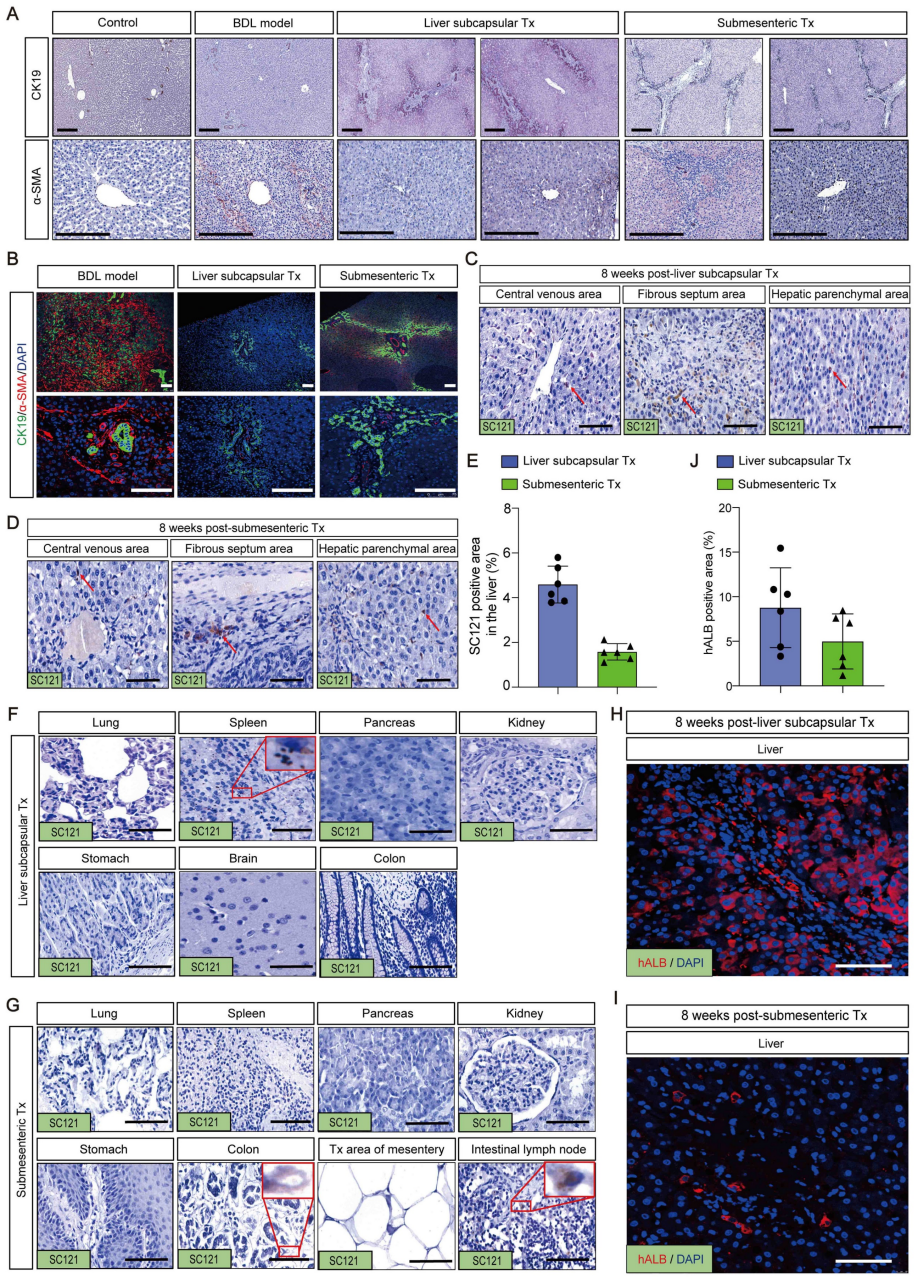
** Transplantation of HBOs stimulated the formation of a new ductular reaction model and inhibited the progression of liver fibrosis. (A)** Immunohistochemical staining of CK19 and α-SMA in liver tissues. Scale bar = 300 μm. **(B)** Immunofluorescence double staining for CK19 and α-SMA. Nuclear DAPI staining in blue. Staining for α-SMA (red) and CK19 (green) were combined. CK19 was found around the periphery of α-SMA positive fibrotic area in the HBO Tx groups, while the BDL model group showed the opposite pattern. Scale bar = 100 μm. **(C-D)** Representative immunohistochemical image of SC121-positive cells in different regions of the liver after HBO transplantation. The results showed that SC121 labeled human cells homing to the liver. Scale bar = 100 μm. **(E)** Ratio of SC121 positive area to the whole liver tissue area. All data were represented as the mean ± SD. ^*^P<0.05 was considered significant. **(F-G)** After HBO transplantation, representative immunohistochemistry imaging of SC121-positive cells in main organs. Scale bar = 100 μm.** (H-I)** The monkey liver tissue was immunofluorescence stained with hALB antibody, and it was identified that human cells retained or homing into the liver contained hepatocytes. Scale bar = 100 μm. **(J)** hALB immunofluorescence intensity quantification using IPP software. Six visual fields were chosen at random. All data were represented as the mean ± SD. ^*^P<0.05 was considered significant. Control, untreated healthy monkey; BDL model, bile duct ligation fibrosis model group without HBO transplantation; Liver subcapsular Tx, liver orthotopic transplantation group; Submesenteric Tx, heterotopic submesenteric transplantation group.

Given that liver fibrosis and ductular response are two of the most prevalent clinical disorders, immunohistochemistry was used to identify the expression of α-SMA and CK19. The results of the staining revealed that in the control group, CK19 was solely expressed in the regular interlobular bile duct in the portal area. In the BDL model group, the CK19-positive ductular reaction (DR) pattern showed a fully developed tubular bile duct-like structure in the fibrous septum, whereas in the liver subcapsular Tx and submesenteric Tx groups, more atypical, small, chain-like bile duct structures formed in the fibrous septum and took up the majority of the fibrous septum space. The α-SMA immunohistochemistry analysis revealed that in the control group, α-SMA was exclusively expressed in the central vein and portal region. The expression of α-SMA was markedly elevated in the fibrous septum of the BDL model group, and it was not only restricted to the fibrous septum but also extended to the central vein, exhibiting a diffuse distribution. The fibrous septum and tiny luminal structures were the only places where α-SMA expression was observed in the liver subcapsular Tx and submesenteric Tx groups (Figure 5A).

Intriguingly, we used immunofluorescence to co-stain CK19 and α-SMA to examine the expression pattern and link between fibrosis and DR. The fibrous septum of the BDL model group was found to have a significant number of α-SMA-positive proliferative fibroblasts, indicating activation of hepatic stellate cells with signs of liver fibrosis. The fibrotic area also contained CK19-positive bile duct structures with severe peritubular fibrosis. In contrast to the BDL model group, the liver subcapsular Tx and submesenteric Tx groups had a significant number of irregular CK19-positive chain-like tiny bile ducts filled in the fibrous septum, and the CK19-positive area encircled the fibrosis area, with only filamentous positive fibroblasts intermingled (Figure 5B). These findings suggested that the DR expression pattern differed between the HBO transplantation group and the BDL model group, with the submesenteric Tx group showing a more typical pattern of triggering a novel DR response than the liver subcapsular Tx group. We hypothesized that the different expression patterns of DR may predict the different prognoses of liver pathological fibrosis based on the difference in α-SMA positive expression levels.

### HBOs could be safely and efficiently retained or homed to the injured liver after HBO transplantation

To ascertain whether the aforementioned therapeutic effects were brought on by HBOs retaining or homing to the injured liver, we used the antibody SC121 to perform immunohistochemical staining of the liver and other main organs to track the whereabouts of human cells after HBO transplantation. The findings revealed that a significant number of SC121-positive human cells were concentrated in the hepatic sinusoids, pathological fibrous septum and central vein region of the liver, suggesting that the shed cells of HBOs after liver subcapsular transplantation were more likely to remain around the vasculature, while human cells from HBOs following submesenteric transplantation may home to the liver through the blood system (Figure 5C, D). For quantification, we compared the percentage of SC121-positive area in the liver between the two HBO transplantation groups and discovered that the liver subcapsular Tx group retained more exfoliated HBO cells than the submesenteric Tx group, but there was no significant difference (Figure 5E).

In addition, we discovered that a small number of SC121-positive cells in the liver subcapsular Tx group persisted in the spleen, and no exfoliated HBO cells developed in any other organs (Figure 5F). In the submesenteric Tx group, we did not find any SC121-positive human cells in the extensive loose mesenteric structure, and only rare SC121-positive cells were detected in the mesenteric lymph nodes and colon, with no positive expression of SC121 found in other organs (Figure 5G). It is evident that the HBOs in the transplanted monkey mesentery had largely transferred from the mesentery, with most of them homing to the liver. Further immunofluorescence labeling with the human hepatocyte-specific marker hALB was performed on liver slices that had SC121-positive retention or homing to determine whether the positive cells included human hepatocytes. The results demonstrated the presence of human liver cells with positive hALB expression in the liver of the subcapsular and submesenteric Tx groups (Figure 5H-J). It was clear that human hepatocytes, which retained or migrated to the liver following HBO transplantation through the liver *in situ* or submesentery, might have a direct therapeutic impact on the repair of damaged liver.

### MMP family genes might act as possible effectors to facilitate effective retention or homing of HBOs

**Figure 6:**
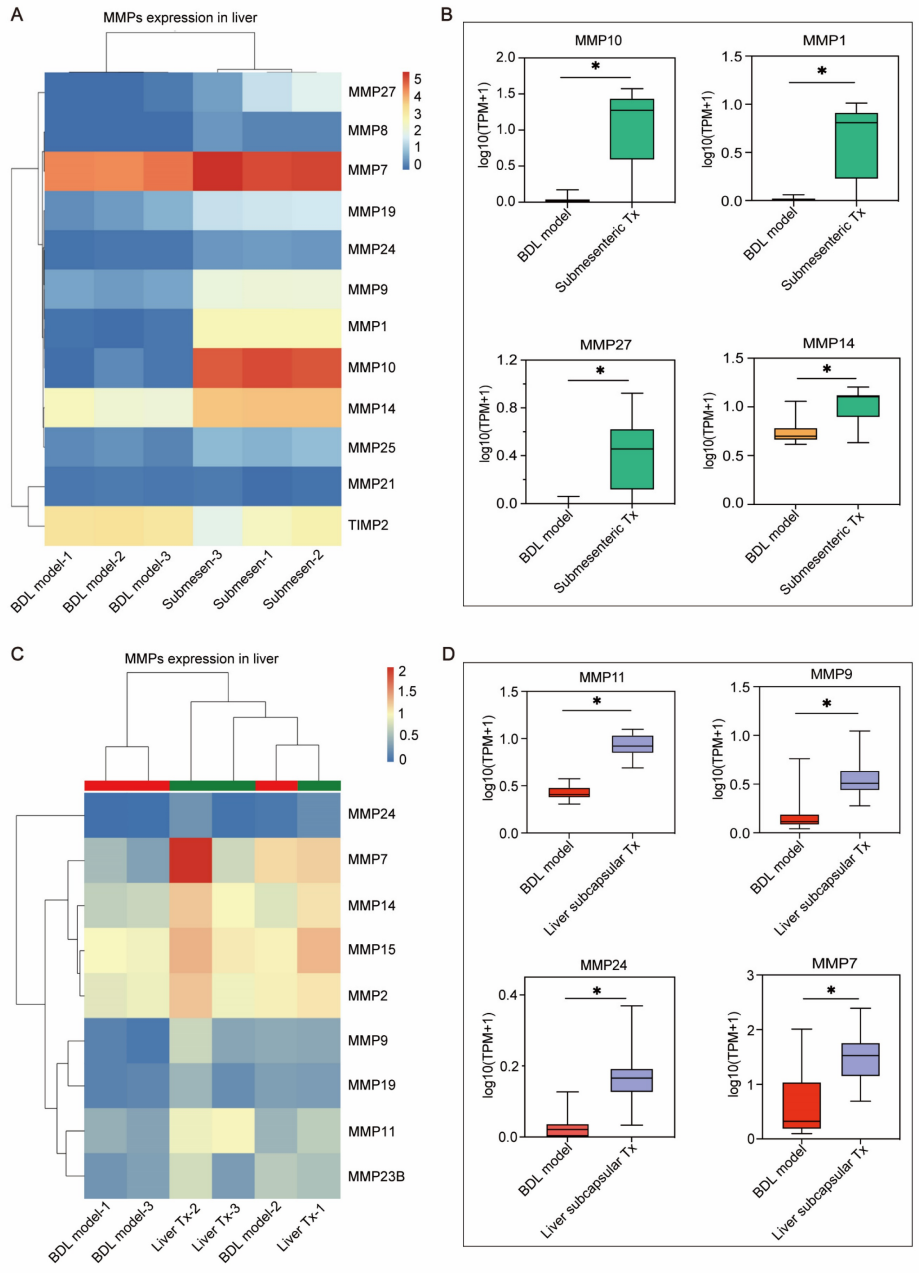
** The MMP family genes significantly up-regulated after transplantation of HBOs may be a potential effector mediating the efficient retention or homing of HBOs. (A)** Heat map of MMPs-related genes within the differentially expressed genes in liver after submesenteric transplantation of HBOs. BDL model-1, bile duct ligation fibrosis model monkey 1; BDL model-2, bile duct ligation fibrosis model monkey 2; BDL model-3, bile duct ligation fibrosis model monkey 3; Submesen-1, heterotopic submesenteric transplantation monkey 1; Submesen-2, heterotopic submesenteric transplantation monkey 2; Submesen-3, heterotopic submesenteric transplantation monkey 3. **(B)** The expression values of the four MMPs genes in the submesenteric Tx group, with the highest up-regulated significance in the MMP family genes have been plotted in log10 1+TPM. BDL model, bile duct ligation fibrosis model group without HBO transplantation; Submesenteric Tx, heterotopic submesenteric transplantation group. **(C)** Heat map of MMPs-related genes within the differentially expressed genes in liver after subcapsular transplantation of HBOs. BDL model-1, bile duct ligation fibrosis model monkey 1; BDL model-2, bile duct ligation fibrosis model monkey 2; BDL model-3, bile duct ligation fibrosis model monkey 3; Liver Tx-1, liver orthotopic transplantation monkey 1; Liver Tx-2, liver orthotopic transplantation monkey 2; Liver Tx-3, liver orthotopic transplantation monkey 3. **(D)** The four MMPs genes in the liver subcapsular Tx group have their expression values presented in log10 1+TPM, with the MMP family gene with the highest up-regulated significance. BDL model, bile duct ligation fibrosis model group without HBO transplantation; Liver subcapsular Tx, liver orthotopic transplantation group.

Based on the previously mentioned RNA-seq results (Figure 3E, F), the genes in MMP family were considerably upregulated in the transplanted livers of HBOs compared to the BDL model group. This gene family attracted our interest because MMPs have been shown to be key players in promoting cell metastasis. Furthermore, we searched the MMP family among the differentially expressed genes and discovered that the submesenteric Tx group had higher levels of MMP1, MMP7, MMP8, MMP9, MMP10, MMP14, MMP19, MMP21, MMP24, MMP25 and MMP27, and the expression of TIMP2, a protease inhibitor (TIMP), was downregulated (Figure 6A). In contrast, the liver subcapsular Tx group had more significant levels of MMP24, MMP7, MMP14, MMP15, MMP2, MMP9, MMP19, MMP11 and MMP23B (Figure 6C). Additionally, the four MMP family members that were most markedly increased in the submesenteric Tx group were MMP10, MMP1, MMP27 and MMP14 (Figure 6B). MMP11, MMP9, MMP24 and MMP7, on the other hand, were considerably higher in the subcapsular Tx group than in the BDL model group (Figure 6D). These findings together suggested that robust expression of the MMP family genes may be connected with both the targeted homing of submesenteric transplanted HBOs to the injured monkey liver and the ability of HBOs transplanted *in situ* in the liver to be retained and survive at the transplantation site.

## Discussion

Although primary hepatocyte transplantation therapies and bio-artificial livers have emerged in response to the urgent demand for liver transplantation replacement therapies, these treatments are typically only employed as short-term liver transplantation alternatives 24. HBOs, a state-of-the-art biomedical technology, provide tremendous application opportunities in the field of regenerative medicine due to their ability to replicate the biological make-up and function of hepatobiliary cells *in vivo*. However, HBO transplantation is still in its early stages at the moment. The first evidence indicating that HBOs can be used for transplantation dates back to 2013, when Huch et al. implanted bile duct organoids derived from LGR5+ endodermal stem cells into nude mice. The implanted bile duct organoids were able to develop into hepatocytes and multiply in the host liver 25. Additionally, liver organoids made from primary human hepatocytes were implanted into damaged livers of immunosuppressed mice, and the organoids survived for 90 days and had an 80% implantation rate 11. These findings offer experimental evidence in support of the use of HBOs in protection/prevention settings. Nevertheless, there are no comprehensive preclinical investigations on the use of HBOs through various transplantation routes in nonhuman primate models.

The present study was the first to evaluate the safety and effectiveness of different transplantation routes of optimally induced HBOs in a nonhuman primate model of liver fibrosis based on the optimized HBO differentiation technology developed by our team in the early stage 21.

Our optimally induced HBOs, generated *in vitro* for approximately 35 days, were able to create hepatobiliary symbiosis organoids with a mature liver and biliary cell phenotype and function. The upper layer of HBOs resembled a tubular or gallbladder, while the lower layer resembled a polygonal liver cell and contained a CD31-positive vascular endothelial structure. It has recently been reported that islet organoids 26 and heart organoids 27 co-cultured with endothelial cells can induce neovascularization in mice, promote the vascularization of organoids in the body, and help organoids better adapt to the host environment after transplantation. Without co-culture with endothelial cells, the HBOs created in this study were able to acquire homologous endothelial cells in the hepatobiliary symbiosis system, opening the door to long-term maintenance of HBOs and survival after transplantation. Additionally, the HBOs we prepared here had a relatively well-developed lipid storage function, and ALB secretion peaked between Days 34 and 36 of HBO culture. Accordingly, we chose HBOs cultivated on the 35th day of *in vitro* differentiation for the *in vivo* transplantation experiment.

The common bile duct ligation, portal vein, and right renal vein shunt operation plan has reportedly been utilized to create a monkey model of acute liver failure 28. In our *in vivo* investigation, partial common bile duct ligation rather than a vein shunt was employed to prevent experimental animals' blood ammonia levels from sharply increasing and causing hepatic encephalopathy and sudden animal death. Additionally, the surgically created liver fibrosis model for monkeys could most effectively avoid the negative effects of extrahepatic toxicity brought on by drug models on the function of other organs, making it a better choice as an animal model construction scheme to assess the safety of HBO transplantation. Subsequently, HBOs cultured *in vitro* for 35 days were transplanted into monkey models with cholestatic hepatic fibrosis via liver subcapsular and submesenteric transplantation routes to evaluate their safety and efficacy.

In terms of safety evaluation, no abnormal changes were found in the renal function sensitive indicators UREA and CREA, the cardiac function indicators CK and LDH, the blood coagulation status indicators APTT, PT, and Fbg, and the routine blood indicators NEUT and LYMPH after HBO transplantation at various time points. There were also no cases of organ embolism. Human cell markers revealed that the majority of shed cells from HBOs transplanted into the body resided in the injured liver, with only a small number of cells found in the spleen, colon, and intestinal lymph nodes, indicating that HBOs had strong targeted homing ability. Undifferentiated hiPSCs can generate teratomas in macaques 29, and 2 months has been noted as the high-risk time point for teratoma formation in hiPSCs 30. We performed a histopathological examination of major organs 2 months after HBO transplantation and found no evidence of teratoma formation, indicating that HBO transplantation is safe and suitable for *in vivo* use.

To evaluate the effectiveness, we detected changes in the expression of the liver injury markers AST and ALT as well as the bile duct injury markers ALP and GGT in monkey peripheral blood. We discovered that AST and ALT in the liver subcapsular Tx group increased transiently after 1 week of transplantation due to liver injury caused by HBO implantation and then quickly returned to low levels. On the other hand, the fluctuations in AST and ALT in the submesenteric Tx group were small within 8 weeks of transplantation. When the two transplantation groups were compared, the submesenteric Tx group demonstrated more obvious liver protection in the acute phase within 2 weeks of transplantation, while the liver subcapsular Tx group illustrated better liver repair potential than the submesenteric Tx group in the subacute phase after 4 weeks of transplantation. Moreover, after 4 weeks of transplantation, both the liver subcapsular Tx and submesenteric Tx groups had a lower change rate of ALP and GGT than the model group, indicating that HBO transplantation may promote bile duct repair. Given that compensatory bile duct dilatation is expected to take place in the later stages of the bile duct ligation model, abdominal ultrasound examination results in monkeys revealed that the inner diameters of the common bile duct and left hepatic duct showed a decreasing trend compared with the model group at 8 weeks after HBO transplantation, which represents the mending effect of HBOs on the liver and bile duct from the side. Furthermore, we used CT to examine the entire liver and discovered that the degree of intrahepatic cholestasis was clearly relieved after HBO transplantation. This suggested that HBOs may reduce cholestasis and improve the state of the injured liver, which could ease compensatory bile duct and hepatic duct dilatation. Considering that cholestasis may cause hepatosplenomegaly, we dissected and quantitatively compared the size of the liver and spleen of monkeys in each group and confirmed the effectiveness of HBO transplantation in inhibiting splenomegaly. With the progression of cholestasis, liver fibrosis and cell apoptosis have emerged as important pathological processes of cholestatic liver injury 31. We used Masson, Sirius Red, and TUNEL staining methods to confirm that HBO transplantation can effectively reverse liver fibrosis and cell apoptosis and that the therapeutic effect in the liver subcapsular Tx group is superior to that of the submesenteric Tx group.

Furthermore, we discovered that the anti-fibrotic effects of HBO transplantation might take place through a combination of mechanisms. The high expression of CTSV in LSECs following HBO transplantation may help to slow the development of hepatic sinusoidal capillarization and liver fibrosis, as was discovered for the first time in our work. The relationship between CTSV and liver fibrosis is currently not covered in other literature. It is known that capillarization of the sinusoids is strongly correlated with the progression of chronic liver diseases and may act as an initiator for the development of liver fibrogenesis 32. The most remarkable phenotypic change of hepatic sinusoid capillarization is the loss of fenestrae, also called defenestration, associated with the formation of a basement membrane on the abluminal surface of LSECs, which is the prelude to fibrosis 33, 34. Since collagen XVIII is a basement membrane constituent that is mainly deposited in liver sinusoids 35, its overexpression may contribute to sinusoidal capillarization, a morphological and pathophysiological hallmark of liver fibrosis. According to research, downregulation of CTSV may be related to the onset of the dermal fibrotic response in systemic fibrosis 36. CTSV also degrades collagen XVIII, and its absence results in fibrosis due to the reduction in collagen dissolution activity. Based on the evidence and research above, we inferred that transplantation of HBOs can reverse the capillarization of liver sinuses by promoting the release of CTSV expression in LSECs, inducing the breakdown of collagen XVIII, and ultimately repairing liver damage and anti-fibrosis.

Another novel finding in our study was that transplanted HBOs could retain or home to the injured liver and initiate a new pattern of DR to reduce the degree of liver fibrosis. There are currently two perspectives on the study of DR: 1) DR is a reparative response to liver cells within the context of liver injury 37 and 2) DR and liver fibrosis are two associated pathological phenomena, and DR can indirectly lead to hepatic fibrosis 38. As shown by our results, the occupied positions of activated fibroblasts and activated hyperplastic bile ducts in the fibrous septum of the liver in the HBO transplantation groups and BDL model group were clearly different, and the difference in DR was significant. The DR form in the BDL model group was mostly a hyperplastic small bile duct with a regular lumen structure, whereas the DR form in the HBO transplantation groups was mostly an irregular hyperplastic small bile duct with no definite lumen. The three main forms of DR that have been identified thus far are type I DR, which refers to a bile duct with a regular lumen structure and is the form of DR that was observed in the model group. Type II DR was an incomplete bile duct-like structure with no distinct morphology, similar to the phenomenon observed in the HBO transplantation groups. Type III DR, which consists of bile duct epithelium and liver oval cells forming a hyperplastic bile duct, is more common after liver sub-mass necrosis, which was not found in our study 39. Following existing DR reports, we believe that the different DR morphologies between the BDL model and HBO transplantation groups reflect the different statuses after liver injury.

In addition, in the HBO transplantation groups, we discovered a new pattern in the locational relationship between DR and positive fibrosis expression. The fibrosis area surrounded the DR-positive area in the BDL model group, but there was a new expression pattern of DR surrounding the fibrosis area in the HBO transplantation groups, and the surrounding fibrosis area was limited to filamentous and punctate cells. This phenomenon was more typical in the submesenteric Tx group. It has been reported that damaged hepatocytes and bile cells activate the rapid initiation of DR as a repair measure for the liver to quickly respond to injury 40. We hypothesized that there might be a dynamic relationship between the two conditions by contrasting the expression and localization patterns of DR and hepatic fibrosis in our study. A recent study demonstrated that the contact distance between the interstitial cell population and bile duct cells can flexibly regulate bile duct proliferation 41. Because it is impossible to accurately reveal the dynamic pathological process in the liver by simply using animal sections at a specific time point, we will attempt to use our HBO as a tool to further explore the dynamic relationship between DR activation and interstitial cells to answer the questions raised by this study.

We also discovered that human ALB-expressing cells were integrated into the liver of the HBO transplantation groups, showing that HBOs transplanted into the liver can reside *in situ* in the liver and that detached cells from HBOs transplanted into the mesentery can home to the liver and exhibit fairly matured hepatocyte activity, but the mechanisms of cell residency and homing are unknown. Therefore, we performed transcriptional sequencing on the liver and discovered a set of MMP genes that were noticeably upregulated in the liver of HBO Tx monkeys. According to studies 42, 43, the MMP gene plays a crucial supporting role in helping mesenchymal stem cells (MSCs) pass the vascular endothelium and basement membrane barrier. Collagen fibers in the basement membrane can be broken down by MMP2 44 and MMP9 45, which are crucial for the migration of MSCs. In our work, we discovered that MMP2 and MMP9 expression was considerably upregulated in the HBO transplantation groups in addition to the co-upregulation of a set of MMP family genes, including MMP10, MMP1, MMP27, MMP14, MMP11, MMP24 and MMP7. One of the possible explanations for the promotion of HBOs retaining or homing to the injured liver of the monkey is the synchronization of numerous MMP components. To identify additional potential targets mediating HBO retention or homing, we will carry out a more thorough study and verification of the monkey liver transcriptome results in the future.

In conclusion, we validated that HBOs transplanted by the liver subcapsular and submesenteric routes are safe and effective by providing a systematic comparative analysis and partially revealed the liver protective mechanism of HBOs after transplantation. The current study did, however, have certain shortcomings. Despite some tests revealing patterns, the high standard deviations and small sample size from this nonhuman study prevented us from yielding statistically significant results. For a comprehensive understanding of the microscopic mechanism by which HBOs repair the liver, more research is needed. Collectively, our findings suggest that further clinical research on optimized HBOs is feasible and may represent a potential avenue for the protection/prevention of liver diseases.

## Materials and Methods

### Cell line and culture conditions

Cell culture was performed with the hiPSC line UC (passages 30 to 37), purchased from Beijing Saibei Biotechnology Company Ltd, Beijing, China (Table S5). Undifferentiated hiPSCs were cultured on qualified Matrigel Basement Membrane Matrix Growth Factor Reduced (Corning, NY, USA)-coated 6-well plates (Thermo Fisher Scientific, Waltham, MA, USA) and maintained in mTeSR medium (STEMCELL Technologies, Vancouver, BC, Canada) at 37 °C with 5% CO_2_ and 95% air. The medium was replaced every 24 h.

### Differentiation of hepatobiliary organoids

Differentiation of hepatobiliary organoids (HBOs) began after at least 5 passages of hiPSCs *in vitro*. The specific differentiation scheme was performed as previously described 21 with appropriate modifications. Briefly, hiPSCs were dissociated into clumps using Accutase (STEMCELL Technologies, Vancouver, BC, Canada) and passaged evenly on a fresh 6-well plate precoated with Matrigel. When the cells reached ∼90% confluency, the medium was replaced with RPMI 1640 containing 2% B27 supplement minus insulin (B27-) (Gibco, Grand Island, NY, USA), 1% penicillin/streptomycin (Gibco, Grand Island, NY, USA), 100 ng/mL activin A and 10 ng/mL BMP4 (PeproTech, Rocky Hill, NJ, USA) for 5 h. Then, 5% mTeSR (STEMCELL Technologies, Vancouver, BC, Canada) was added to the above RPMI 1640/(B27-) culture system and continually cultured for 2 days. To induce the cells to the oriented endoderm stage, the medium was replaced with RPMI 1640 containing 2% B27-, 1% penicillin/streptomycin, 5% mTeSR and 100 ng/mL activin A to continue cultivation for another 2 days. Next, the medium was replaced with RPMI 1640 containing 2% B27 (Gibco, Grand Island, NY, USA), 1% penicillin/streptomycin, 5% mTeSR, 20 ng/mL BMP2 and 30 ng/mL FGF4 (PeproTech, Rocky Hill, NJ, USA) for 5 days, followed by RPMI 1640 containing 2% B27, 1% penicillin/streptomycin, 5% mTeSR, 20 ng/mL HGF and 20 ng/mL KGF (PeproTech, Rocky Hill, NJ, USA) for another 6 days to induce hepatic progenitor cell formation. Following this, HCM (Lonza, Basel, Switzerland) contained 10 ng/mL oncostatin M (PeproTech, Rocky Hill, NJ, USA), 1% penicillin/streptomycin, 0.5 μM dexamethasone (Sigma‒Aldrich, St. Louis, MO, USA) and 10% cholesterol^+^ MIX (ProbeChem, Shanghai, China) was used to culture for another 20 days. Subsequently, the culture medium was replaced with high glucose DMEM (Gibco, Grand Island, NY, USA) containing the same concentration (1:1,000) of ascorbic acid, transferrin, insulin, gentamicin/amphotericin-B (Lonza, Basel, Switzerland), 0.2% fatty acid free bovine serum albumin, 10 ng/ml OSM and 10% cholesterol^+^ MIX for further culture. The culture medium was changed every 24 h. The differentiation protocol is shown in Figure S2A. The optimally induced HBOs that were selected for transplantation expressed hepatobiliary-specific markers and exhibited excretion functions (Figure S2B-G).

### Nonhuman primate modeling and HBO transplantation

Animal experiments were conducted in accordance with the regulations of the Institutional Animal Care and Use Committee of Sichuan Green-house Biotech Co., Ltd. (Sichuan, China), approval number: IACUC- B2020016-P-01. Three- to four-year-old male cynomolgus monkeys weighing between 3-5 kg (purchased from Sichuan Green-house Biotech Co., Ltd, Sichuan, China) were used as recipients (Table S6). Cynomolgus monkeys were given a continuous immunosuppressive treatment (tacrolimus + mycophenolate mofetil + methylprednisolone) 5 days before modeling. Each monkey was subjected to general anesthesia with 2% sevoflurane inhalation anesthesia after intramuscular injection of atropine sulfate (0.05 mg/kg) and Zoletil (0.1 ml/kg). Next, the lower part of the xiphoid process and the middle part of the upper belly of the umbilicus were used to make a longitudinal surgical incision and expose the abdomen layer by layer. After that, it was confirmed that the size, texture and color of the liver were normal, we used a tissue collector to take a small piece of liver tissue (5-8 mm^3^) as the control sample for subsequent histopathological analysis. After laparotomy, we dissociated the hepatic portal vein, hepatic artery and common bile duct and then ligated the common bile duct with 2/0 silk thread to narrow the common bile duct by 70% (Figure 1B).

*Liver subcapsular transplantation of HBOs.* Within 30 min after successful modeling, a tissue puncture needle was fixed to a 1 mL syringe, and mature HBOs on Day 35 (total 2×10^6^ cells/kg) were gently lifted onto the puncture needle with tweezers. The syringe was withdrawn to allow HBOs to stay fully inside the puncture needle, and the piston handle of the syringe was inserted into HBOs (total 2×10^6^ cells/kg) at 5 transplantation sites under the liver capsule. The liver incision was closed with a suture needle to prevent intraoperative liver hemorrhage. Finally, the abdominal cavity was closed layer by layer after it was confirmed that there was no obvious bleeding.

*Submesenteric transplantation of HBOs.* After successful modeling, mature HBOs on Day 35 (total 2×10^6^ cells/kg) were transplanted into 6 randomly selected sites in the colon mesentery. HBOs were removed from the culture medium with tweezers, grafted in the transplantation sites of the mesentery (see Figure 1B), and then sutured with silk thread.

### Hematology and Biochemical assays

Venous blood was harvested from experimental monkeys before modeling as a baseline, and blood sampling continued weekly from 1-8 weeks after HBO transplantation. Blood samples were centrifuged at 4000 r/min and 20 °C for 10 min and then evaluated in an Automatic Biochemical Analyzer (Hitachi, Tokyo, Japan). All indicators are listed in Table S4.

### Abdominal ultrasonographic examination

After anesthetization with a mixture of sevoflurane and oxygen, the monkeys were placed in dorsal recumbency, the abdomen was shaved, and sonographic coupling gel was applied. Transabdominal ultrasound of the liver was performed by experienced sonographers using a transportable B-mode ultrasound device (Toshiba Medical Systems, Tokyo, Japan) with a 3.5 MHz ultrasonic transducer. The liver was visualized in the sagittal and transverse planes with the transducer positioned at the ventral midline caudal to the xiphoid, directing it anteriorly.

### CT scan and imaging analysis

An abdominal CT scan was obtained using a 16-row multislice CT (G.E., Milwaukee, WI). The standard slice was 5 mm thick. CT exams were reviewed by two board-certified radiologists separately and then together to come to a consensus. The radiologists were blinded to the purpose and design of the study.

### TUNEL assay

The liver tissue sections were deparaffinized in xylene, gradually rehydrated in decreasing concentrations of ethanol, and then stained with a TUNEL staining kit (KeyGEN, Nanjing, China) according to the instructions to detect apoptosis. The images were obtained using a Super Resolution Tissue Microscope (Leica, Wetzlar, Germany), and the numbers of TUNEL-positive cells in 5 randomly selected fields were counted.

### Albumin ELISA

Albumin (ALB) secreted from HBOs was determined by a human ALB-ELISA kit (Proteintech, Wuhan, China). After 26 days of HBO differentiation *in vitro*, the culture supernatants were collected every 2 days, and the secretion of ALB was detected at different time points according to the manufacturer's instructions.

### Flow cytometry

Samples were dissociated into single cells with Tryple (Gibco, Grand Island, NY, USA) at 37 °C. After centrifugation, the cells were fixed with 4% paraformaldehyde (PFA) at room temperature (RT) for 15 min, centrifuged and incubated in PBS (Solarbio, Beijing, China) containing 10% BSA (Sigma‒Aldrich, St. Louis, MO, USA), 10% normal goat serum (BIORIGIN, Beijing, China) and 0.3% Triton X-100 (Sigma‒Aldrich, St. Louis, MO, USA) for 1 h. Next, the cells were incubated with primary antibodies at RT for 1.5 h and then with secondary antibodies at RT for 30 min in the dark. Analysis was performed using a FACSC auto flow cytometer (BD Biosciences, San Jose, CA, USA). All antibodies are listed in Table S3.

### Histology and Immunofluorescence

For frozen sections, HBOs were fixed in 4% PFA and then dehydrated in 30% sucrose solution at RT for 24 h, embedded in OCT medium (SAKURA, Tokyo, Japan) and cut into 8 μm-thick slices. Dehydrated monkey tissue samples were fixed in paraffin and divided into 5 μm slices. Frozen sections were rinsed in PBS and then fixed in 4% PFA for 15 min, while paraffin sections were gradually deparaffinized and rehydrated followed by subsequent staining experiments.

Hematoxylin-eosin (HE), Masson, Oil Red O (Solarbio, Beijing, China) and Sirius red (Abcam, Cambridge, MA, USA) staining were performed in accordance with the manufacturer's instructions.

For immunohistochemical staining, sections were subjected to antigen repair and subsequently blocked in 10% normal goat serum, 5% BSA and 0.3% Triton X-100 diluted in PBS at RT for 1 h. Primary antibodies were incubated overnight at 4 °C, washed 3 times with PBS and then incubated with secondary antibodies at 37 °C for 30 min. After staining with DAB (ZSGB-BIO, Beijing, China) and hematoxylin, sections were rehydrated and transparent again and finally mounted and dried at RT overnight. All antibodies are listed in Table S2.

For immunofluorescence staining, sections were washed 3 times with PBST and then blocked in 10% normal goat serum, 5% BSA and 0.3% Triton X-100 diluted in PBST at RT for 1 h. Primary antibodies were incubated overnight at 4 °C, and secondary antibodies were incubated for 1 h at room temperature. Sections were stained with DAPI (Solarbio, Beijing, China) for nuclear staining and mounted using mounting medium. All antibodies are listed in Table S1.

All histological images were acquired using an Aperio VERSA Digital Pathology Scanner (Leica, Wetzlar, Germany), and immunofluorescence images were captured with laser scanning confocal microscopy using a Leica TCS SP8 confocal microscope (Leica Microsystems, Wetzlar, Germany). Images were analyzed with NIH ImageJ software.

### RNA extraction and RNA-seq analysis

Briefly, total RNA was extracted from monkey livers using TRIzol^®^ Reagent (Invitrogen, Carlsbad, CA, USA) according to the manufacturer's protocol and qualified using a NanoDrop 2000 spectrophotometer (Thermo Fisher Scientific, Waltham, MA, USA) and Agilent 2100 bioanalyzer (Agilent Technologies, Santa Clara, CA, USA). mRNA was enriched using oligo (dT) magnetic beads and reverse transcribed to cDNA. A TruSeqTM RNA Sample Preparation Kit (Illumina, San Diego, CA, USA) was used to prepare the RNA sequencing libraries. Finally, the cDNA library was pooled, and paired-end 150 bases were sequenced on a NovaSeq 6000 (Illumina, San Diego, CA, USA). Fastp (https://github.com/OpenGene/fastp) and the default parameters matching the end reading of the original trim and quality control were used. The clean reads were aligned to the reference genome by HISAT2 (http://ccb.jhu.edu/software/hisat2/index.shtml) software. The mapped reads of each sample were assembled by StringTie in a reference-based approach 46.

### Differential expression analysis and bioinformatics analysis

To identify differentially expressed genes (DEGs) between the two groups ("Liver subcapsular Tx vs. BDL Model" group and "Submesenteric Tx vs. BDL Model" group), the expression level of each gene was calculated based on the transcripts per million reads (TPM) method. Gene abundance was quantified using the RSEM tool (http://deweylab.biostat.wisc.edu/rsem/). DESeq2 was used to perform differential expression analysis. |log2 (foldchange)| > 1 and *P* value < 0.05 were set as the thresholds for significant DEGs. A volcano map was drawn to show the DEGs (www.bioinformatics.com.cn). The vertical coordinate is displayed as the negative log10 (*P* value) of the T test significance test, and the abscissa is the log2 multiple change value (Fc). Each node represents a gene. The red node indicates that the gene meets the DEG screening conditions (|log2FC| >1 and *P* value <0.05), the green node indicates that the gene only meets |log2FC| >1, the blue node indicates that the gene only meets *P* value <0.05, and the gray node indicates that the gene does not satisfy |log2FC| >1 or *P* value <0.05, and there is no significant difference in this gene. The DEGs obtained in the "Liver subcapsular Tx vs. BDL Model" group and "Submesenteric Tx vs. BDL Model" group were sorted in descending order by |log2FC|. The top 10 genes upregulated after HBO liver subcapsular Tx and the top 10 genes upregulated after HBO submesenteric Tx were selected and are presented in a bar graph. The expression of MMPs in the liver before and after HBO transplantation is displayed by a cluster heatmap. The significantly altered MMP family genes after transplantation of HBOs were imported into the online tool of the Majorbio Cloud Platform (https://cloud.majorbio.com/page/tools/) to draw a cluster heatmap. The cluster heatmap shows the gene expression level (TPM) through the change in the shade of color.

The CTSV gene expression level was obtained from the tissue cell type and single cell type sections in the Human Protein Atlas (HPA) database (proteinatlas.org) 47. The tissue cell type panel and single cell type sections show the expression of protein-coding genes in human cell types based on bulk RNAseq data and the expression of protein-coding genes in single human cell types based on scRNA-seq, respectively. The uniform manifold approximation and projection (UMAP) cluster plot (https://www.proteinatlas.org/ENSG00000136943-CTSV/single+cell+type/liver), bar plot of the correlation of CTSV with different cell types in the liver, and heatmap of the correlation between CTSV and marker genes of different liver cell types (https://www.proteinatlas.org/ENSG00000136943-CTSV/tissue+cell+type/liver) were downloaded from the HPA database.

### Statistical analysis

Statistical analysis was performed using SPSS 26.0 software, and the results are expressed as the mean ± standard deviation. After the test of normality and congruence of variances, one-way ANOVA was used to compare groups with equal variance, and a nonparametric test was used to compare groups with uneven variance. When the *P* value was <0.05, the differences were regarded as statistically significant. Eventually, GraphPad Prism 8 (GraphPad Software, USA) was used to summarize the data and plot the statistics.

## Supplementary Material

Supplementary figures and tables.
