# Supplementary material for: Hepatobiliary organoids differentiated from hiPSCs relieve cholestasis-induced liver fibrosis in nonhuman primates
Source: Int J Biol Sci. 2024 Jan 21;20(4):1160–79. doi: 10.7150/ijbs.90441 (PMC10878143; doi:10.7150/ijbs.90441)
Supplement: Supplementary file 1 — Supplementary figures and tables. [file ijbsv20p1160s1.pdf]

Hepatobiliary organoids differentiated from hiPSCs relieve cholestasis-induced liver fibrosis in nonhuman primates

Hongmei Li, Jingyi Li, Ting Wang, Ke Sun, Guangrui Huang, Yulin Cao, Fenfang Wu, Anlong Xu

Table of contents

Figure S1 2

Figure S2 3

Figure S3 4

Table S1 6

Table S2 7

Table S3 7

Table S4 7

Table S5 8

Table S6 8

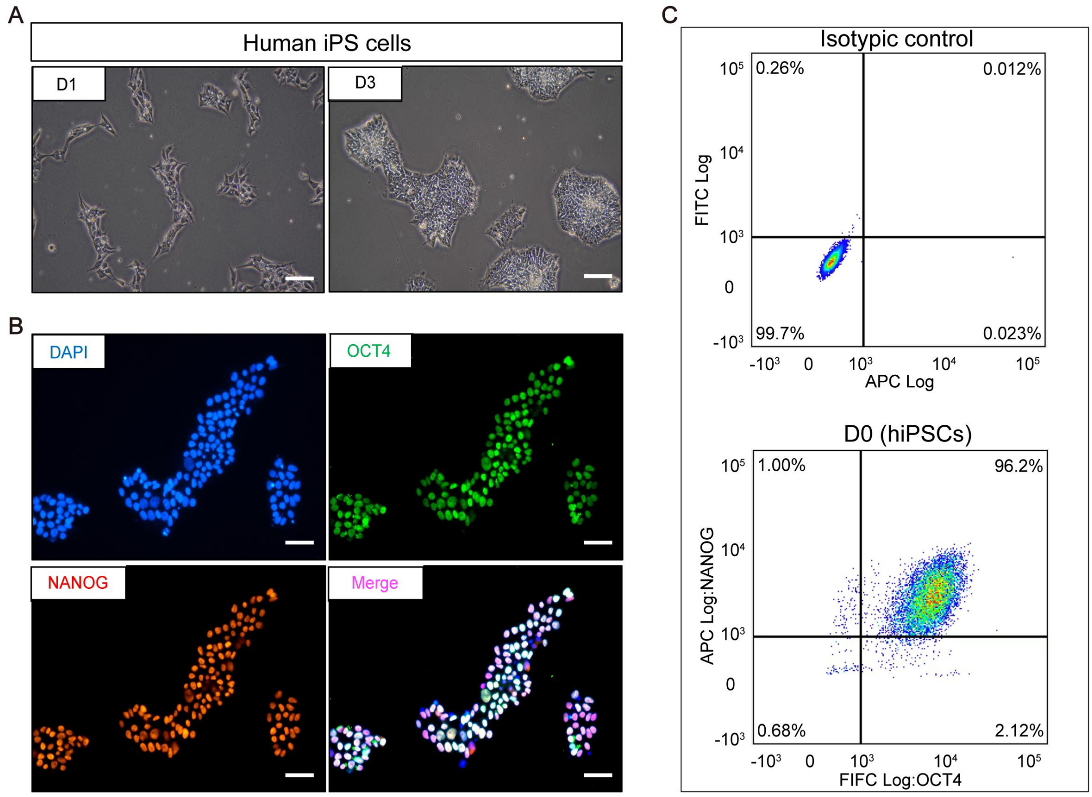

Figure S1. Characterization of human induced pluripotent stem cells.

(A) Bright-field images of cultured human iPS cells. Scale bars: 50μm. **(B)** Immunofluorescence analysis showed the presence of pluripotency markers OCT4 and NANOG. Cells were counterstained with DAPI to visualize cell nuclei. Scale bar: 50 μm. **(C)** Representative flow cytometry analysis of the pluripotency marker expression for hiPSCs cell line. The results showed that the proportion of hiPSCs with double positive expression of OCT4 and NANOG was 96.2%. hiPSCs, human induced pluripotent stem cells; D1, hiPSCs culture for one day; D3, hiPSCs culture for three days.

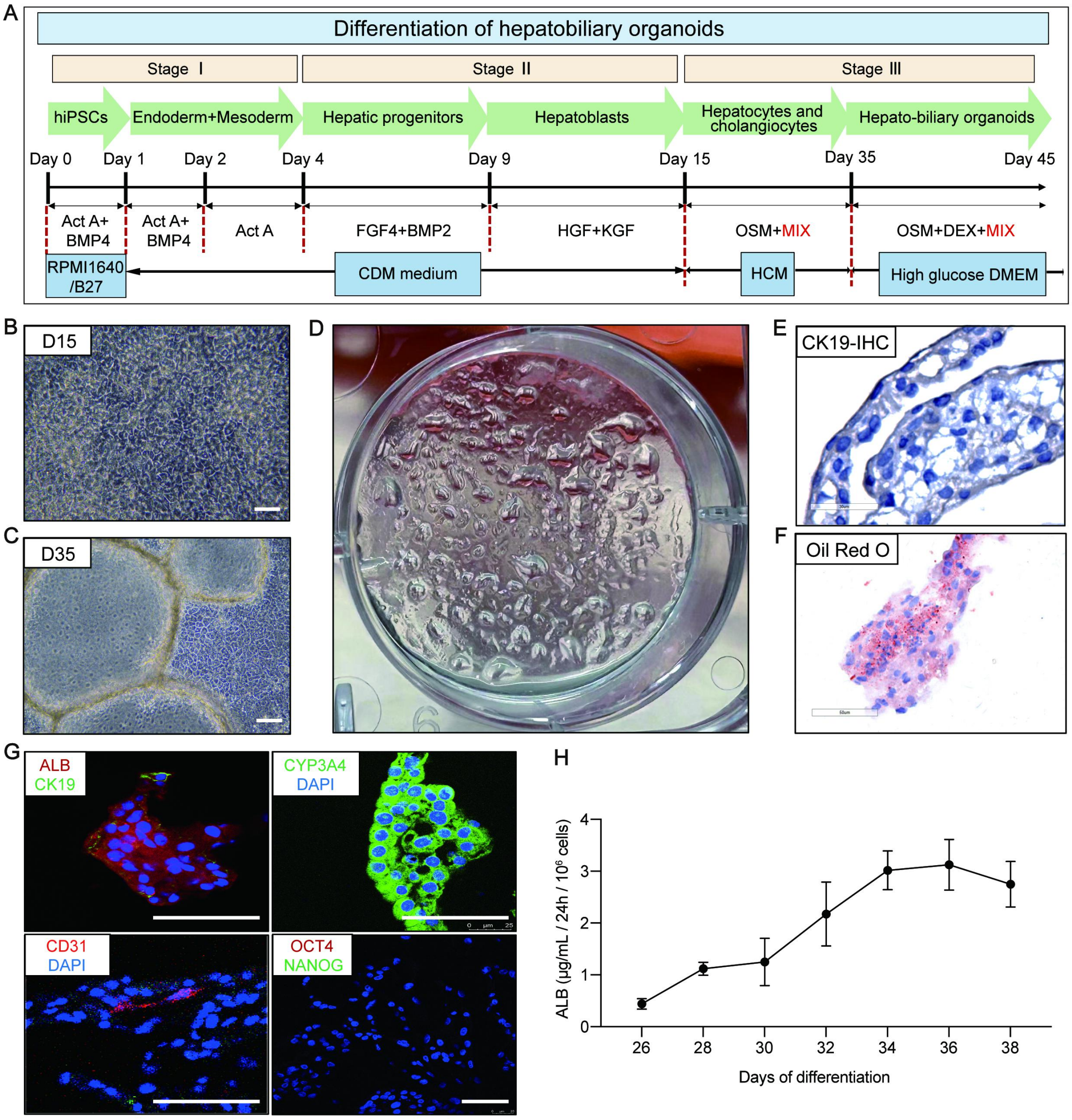

Figure S2. Differentiation and identification of HBOs.

(A) Schematic representation of the differentiation procedure (day 0-45) of hiPS differentiation into HBOs induced by our self-developed differentiation protocol. **(B)** Bright-field image of hiPSCs differentiation to HBOs on day 15. The cells were in the differentiation stage of liver progenitor cells. Scale bars: 50μm. **(C)** Bright-field image of hiPSCs differentiation to HBOs on day 35. At this stage of differentiation, liver cells were distributed in the lower layer of HBOs, and biliary cells were distributed in the upper layer of HBOs, in tubular or vesicular shape. Scale bars: 50μm. **(D)** Representative photo of real HBOs in a six-well plate after 35 days of differentiation. **(E)** Immunohistochemical staining of CK19 showed that HBO contained bile duct like structures with positive expression of CK19. Scale bars: 30μm. **(F)** Oil Red-O staining of the HBOs for lipid accumulation. The results showed that the hepatocytes in HBOs had the similar lipid storage function as the liver cells in the body. Scale bars: 50μm. **(G)** Immunofluorescence staining showed that mature HBOs could simultaneously express mature liver cell markers ALB, CYP3A4, and vascular endothelial structure marker CD31. While all cells in HBOs did not express stem cell multipotent markers OCT4 and NANOG. Scale bars: 30μm. **(H)** From the 26th day of HBOs

differentiation *in vitro*, the supernatant was collected every 2 days, and the expression of ALB protein was measured by ELISA to identify the secretion function of HBOs. HBOs were distinct samples derived from the same hiPSC line with the same differentiation. N = 3 biological replicates, SEM are depicted as error bars.

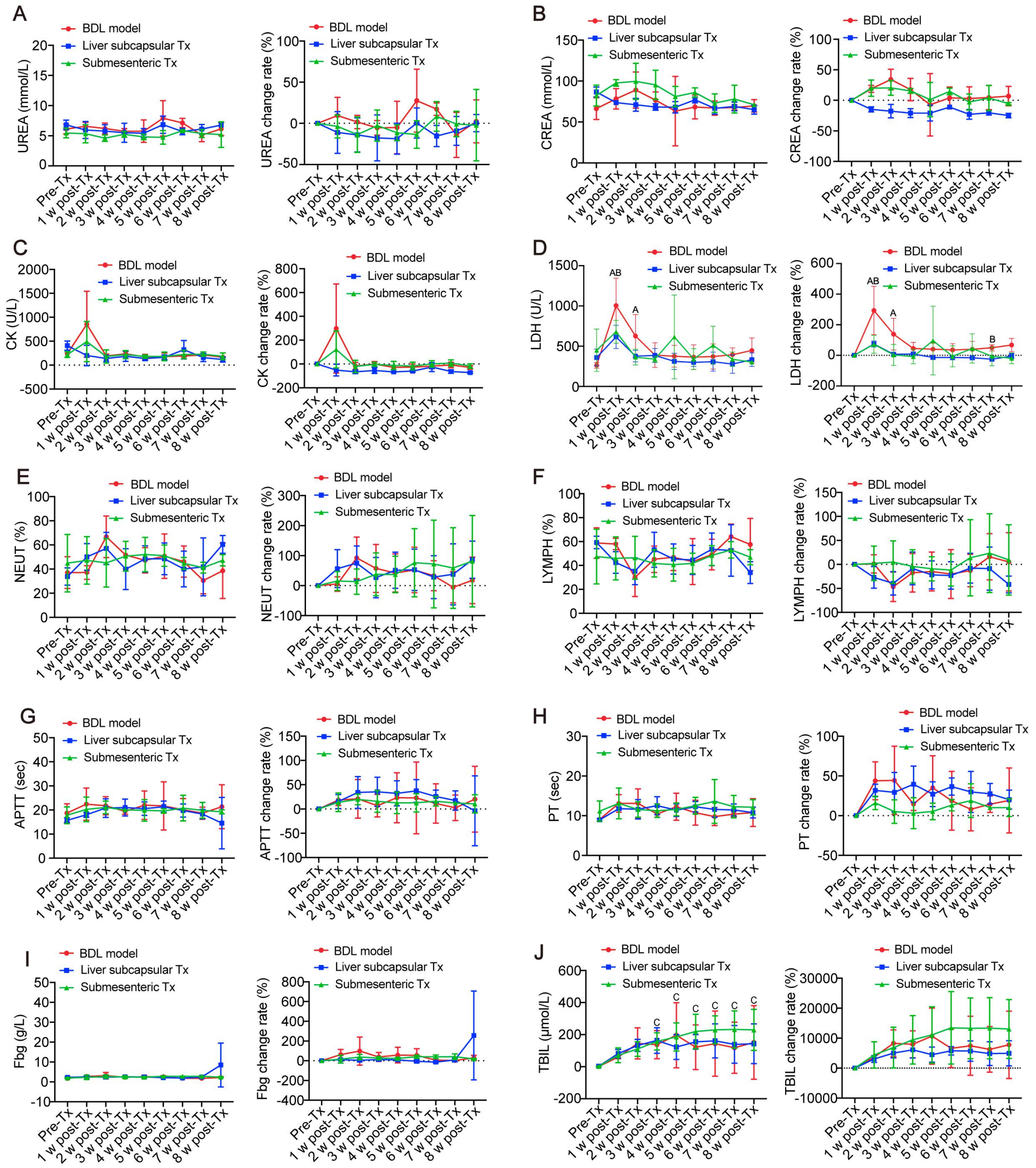

Figure S3. The safety of HBO transplantation was evaluated by the blood biochemistry test.

(A-B) UREA and CREA were measured as biochemical parameters to evaluate renal function after HBO transplantation. **(C-D)** CK and LDH were used to evaluate the safety of heart after HBO transplantation. **(E-F)** Evaluate the blood status of the whole body through the detection of NEUT and LYMPH in the blood routine indicators. **(G-I)** Values of APTT, PT, and Fbg as the three indicators of coagulation function reflect that HBO transplantation is safe for the

blood system. **(J)** Changes of TBIL in nonhuman primates at different time points after HBOs transplantation. UREA: Urea nitrogen; CREA: Creatinine; CK: Creatine kinase; LDH: Lactate dehydrogenase; NEUT: Neutrophil; LYMPH: Lymphocyte; APTT: Activated partial thromboplastin time; PT: Prothrombin time; Fbg: Fibrinogen; TBIL: Total bilirubin. Data were represented as mean ± SEM (n=3 per group). ^{A} indicates P<0.05 post-Tx versus pre-Tx in the model group. ^{B} indicates P<0.05 post-Tx versus pre-Tx in the Liver subcapsular Tx group. ^{C} indicates P<0.05 post-Tx versus pre-Tx in the Submesenteric Tx group. BDL model, bile duct ligation fibrosis model group without HBO transplantation; Liver subcapsular Tx, liver orthotopic transplantation group; Submesenteric Tx, heterotopic submesenteric transplantation group; Pre-Tx, before modeling and transplantation; 1 w post-Tx, 1 week after modeling and transplantation; 2 w post-Tx, 2 weeks after modeling and transplantation; 3 w post-Tx, 3 weeks after modeling and transplantation; 4 w post-Tx, 4 weeks after modeling and transplantation; 5 w post-Tx, 5 weeks after modeling and transplantation; 6 w post-Tx, 6 weeks after modeling and transplantation; 7 w post-Tx, 7 weeks after modeling and transplantation; 8 w post-Tx, 8 weeks after modeling and transplantation.

Table S1. Antibodies used in immunofluorescence analyses.

Target	Host	Supplier	Cat no.	Dilution
OCT4	Rabbit	Cell Signaling	#2750	1:200
NANOG	Mouse	Cell Signaling	#4893	1:1000
ALB	Mouse	Bio-Techne	MAB1455	1:100
CYP3A4	Rabbit	Proteintech	18227-1-AP	1:100
CK19	Rabbit	Proteintech	14965-1-AP	1:400
CD31	Rabbit	Abcam	ab32457	1:50
CD31	Mouse	Abcam	ab9498	1: 500
α-SMA	Rabbit	Proteintech	14395-1-AP	1:200
α-SMA	Mouse	Novus	NBP2-33006	1:250
LYVE-1	Rabbit	Abcam	Ab219556	1:5000
CTSV	Mouse	SANTA CRUZ	sc-32798	1:50
Anti-rabbit IgG (H+L)-Alexa Fluor® 488 Conjugate	Goat	Cell Signaling	#4412	1:500
Anti-mouse IgG (H+L)-Alexa Fluor® 594 Conjugate	Goat	Cell Signaling	#8890	1:500
Anti-mouse IgG (H+L)-Alexa Fluor® 647 Conjugate	Goat	Cell Signaling	#4410	1:500
Goat Anti-Mouse IgG(H+L)-Alexa Fluor 488	Goat	Biorigin	BN20630	1:200
Goat Anti-Rabbit IgG(H+L)-Alexa Fluor 647	Goat	Biorigin	BN20636	1:200
Donkey Anti-Goat IgG H&L / AF555	Donkey	Bioss	bs-0294D-AF555	1:200

OCT4: Organic cation/carnitine transporter 4; ALB: Albumin; CYP3A4: Cytochrome P450 family 3 subfamily A member 4; CK19: Cytokeratin 19; CD31: Platelet and endothelial cell adhesion molecule 1; Lyve-1: Lymphatic vessel endothelial hyaluronan receptor 1; α-SMA: Alpha-smooth muscle actin.

Table S2. Antibodies used in immunohistochemistry analyses.

Target	Host	Supplier	Cat no.	Dilution
CK19	Rabbit	Proteintech	14965-1-AP	1:500
α-SMA	Rabbit	Proteintech	14395-1-AP	1:200
α-SMA	Mouse	Novus	NBP2-33006	1:400
CD31	Mouse	Abcam	ab9498	1:1000
Lyve1	Rabbit	Abcam	Ab219556	1:5000
SC121	Mouse	Takara Cellartis	Y40410	1:750
CTSV	Mouse	SANTA CRUZ	sc-32798	1:50

SC121: also known as STEM121, a marker of human cell engraftment that can be used to differentiate animal from human cells.

Table S3. Antibodies used in flow cytometry.

Target	Host	Supplier	Cat no.	Dilution
OCT4	Rabbit	Cell Signaling	#2750	1:400
NANOG	Mouse	Cell Signaling	#4893	1:800
Anti-rabbit IgG (H+L)-Alexa Fluor® 488 Conjugate	Goat	Cell Signaling	#4412	1:100
Anti-mouse IgG (H+L)- Alexa Fluor® 647 Conjugate	Goat	Cell Signaling	#4410	1:100

Table S4. Relevant indicators in hematological and biochemical tests.

Project	Indication
Liver function	AST, ALT, ALP, GGT, TBIL
Renal function	UREA, CREA
Cardiac function	CK, LDH

Blood coagulation	APTT, PT, Fbg
Blood routine index	NEUT, LYMPH

Table S5. Cell lines.

Name	Supplier	Passage no.
hiPSCs	Beijing Saibei Biological Technology Co. Ltd.	Passage 30-37

Table S6. Organisms.

Name	Supplier	Sex	Age	Body weight	Overall number
Cynomolgus monkeys (Macaca Fascicularis)	Sichuan Green-house Biotech Co., Ltd.	Male	3-4 years	3-5kg	12
